# Studying the Association Between Knowledge of Professionalism and Demographic Characteristics in King Saud University Medical Students

**DOI:** 10.7759/cureus.44241

**Published:** 2023-08-28

**Authors:** Nasser S Alharbi, Abdullah M Alassaf, Abdullah R AlZamil, Bader M Alqarni, Faisal A Alzahrani, Faisal B Alsaif

**Affiliations:** 1 Pediatrics, King Saud University, Riyadh, SAU; 2 Medicine, King Saud University, Riyadh, SAU

**Keywords:** professionalism, medical students, knowledge, gender, age

## Abstract

Objectives

To study the association between the level of knowledge regarding professionalism and demographic characteristics among medical students from years three to five at King Saud University, Riyadh, Saudi Arabia.

Methods

Data for this quantitative observational cross-sectional study were collected using stratified random sampling. The participants included male and female students from years three to five studying at the College of Medicine, King Saud University. Data were collected using a self-administered questionnaire and analyzed using the Student's t-test and a one-way ANOVA test.

Results

The study comprised 112 female (52%) and 103 male (48%) students who completed 215 questionnaires. The mean percentage values of correct answers by females and males were 59.99% and 59.31%, respectively. Statistical analysis revealed no significant difference between the mean percentage of correct answers given by males and females (p=0.684). The mean percentage of correct answers among the 3rd, 4th, and 5th-year medical students was 59.27%, 56.56%, and 62.72%, respectively. Statistical analysis revealed significant differences between the academic year groups (p<0.05), and the grade point average (GPA) groups showed significant differences (p<0.05).

Conclusion

A highly significant association was found between knowledge of professionalism and both academic level and performance among medical students. This suggests that professional perception evolves parallel to acquiring basic science and clinical knowledge.

## Introduction

Professionalism is an old and respectable concept in medicine [1]. The term 'professionalism' is derived from the Latin word 'professus,' meaning to have declared publicly [2]. Nowadays, professionalism is defined as a "set of values and behaviors which determine the extent of moral values between the profession and society." Thus, there is a moral relationship between patients and doctors [3-6]. The public's trust in doctors and healthcare workers makes medicine a sacred and valuable profession; weak professionalism in physicians is an essential cause of medical malpractice and errors [1]. Therefore, professionalism is considered a critical quality for physicians to possess. A study conducted to evaluate the perception of professionalism stated that the values related to professionalism, such as integrity, accountability, responsibility, tolerance, compassion, maturity, and appearance, may vary widely with several factors, such as age, gender, type of healthcare student, level of education, and social background [7]. Many authorities suggest that understanding these factors contributing to professionalism may allow the development of more effective approaches to promoting this quality in medical education [8-10]. Studies should investigate the correlation between the level of knowledge on professionalism and the influencing factors among students. Accordingly, this study was conducted at King Saud University among the students in the College of Medicine, using gender, academic year, and grade point average (GPA), which represents academic performance, as the only feasible variables to measure the students via an electronic-based questionnaire. Measuring professionalism can pose challenges due to the different methods of assessment. Therefore, assessing professionalism in the knowledge aspect is preferable among medical students. Hence, this study aimed to evaluate the association between the level of knowledge of professionalism and gender. Among those medical students, the only ones who finished the curriculum of professionalism in the College of Medicine at King Saud University were 3rd, 4th, and 5th year medical students. Therefore, 1st and 2nd-year medical students were excluded to minimize confounding factors between academic levels, as there is evidence that a focused curriculum in professionalism may improve participant knowledge and overall group behavior [11]. 

Factors contributing to professionalism have been discussed in several studies. A study conducted in Iran showed that gender is a statistically significant factor for the knowledge of professionalism, with female participants exhibiting significantly higher knowledge levels than male participants. However, age and degree showed no significant difference in the level of knowledge [1]. In contrast, a study in America explored factors affecting the perception of professionalism and observed that the perception of professionalism varied most with the level of education, age, and, to a lesser extent, gender and healthcare discipline [7]. 

## Materials and methods

This cross-sectional quantitative observational study conducted at the College of Medicine at King Saud University, Riyadh, Saudi Arabia, between May 2021 and July 2021, was approved by the institutional review board (approval no. E-20-5446). The authors used a questionnaire consisting of two sections: the first section obtained data on the demographic characteristics of the participants and contained three questions on gender (male or female), academic level (3rd, 4th, and 5th), and GPA as class interval choices (<3.5, 3.5 to 3.99, 4 to 4.49, and 4.5 to 5); the second section contained a validated tool that examined participant knowledge in areas related to professionalism and comprised 21 true or false statements with true, false, and I don’t know options. The validity of the questionnaire was analyzed and confirmed by the original author of the tool with a 0.755 Cronbach’s alpha score for the 21 items of the questionnaire [1].

The target study population was medical students at King Saud University from academic years three to five, from both genders. Students from the 1st and 2nd years were excluded from the study as they had not yet gone through the professionalism curriculum at the College of Medicine, King Saud University. The sample size required for the study was calculated using the following equation: n = 2S2 (Zα + Zβ) / d2, where Z is the standard normal score with a 95% confidence interval (CI) (a = 0.05), S is the standard deviation of the variable obtained from a similar previous study (=16) [1], and d (5%) is the maximum acceptable error. The sample size was estimated at 214, with a confidence level of 95% and a 0.05 margin of error. After adding 20% to the sample size to cover the possible non-response rate, 257 random participants were selected using a stratified random sampling technique where the investigator divided the population into six strata based on gender (male or female) and academic year (3rd, 4th, and 5th years) and randomly selected the final subjects proportionally from each stratum. Electronic-based questionnaires were distributed to these random participants via university emails. 

The study variables were gender, academic year, and GPA, and the outcome variable was the percentage of correct answers from the 21 questions in the tool. The data were analyzed using SPSS Statistics version 24.0 (IBM Corp., Armonk, NY, USA). An independent Student's t-test was used to analyze the difference in the mean percentage of correct answers between males and females, and a one-way ANOVA test was used to analyze the difference between academic year groups and GPA groups. A p-value less than 0.05 was considered statistically significant.

All responses were anonymous and identified by codes to ensure data confidentiality for participants. Electronic consent was obtained from participants upon presentation of the questionnaires. Ethical considerations were taken into account during the dissemination of data and publication of results. The original author of the tool provided consent for its use in this study.

## Results

Table 1 shows the 215 responses analyzed for the study: 112 students were female (52%), and 103 were male (48%). The table also shows the distribution of students in the 3rd, 4th, and 5th academic years as 74 (34%), 65 (30%), and 76 (35%), respectively. The GPA variables among the students for class intervals were <3.5, 3.5 to 3.99, 4 to 4.49, and 4.5 to 5, respectively, and the number of responses were 6 (2%), 32 (15%), 94 (44%), and 83 (39%), respectively. Based on gender, the mean percentages of correct answers by males and females were 59.36% (SD=12.3) and 59.99% (SD=12), respectively, and the mean difference between males and females was 0.63%. The t-test revealed no statistically significant difference, as the p-value of 0.684 was higher than 0.05. For the academic year variable, the mean percentage of correct answers given by 3rd, 4th, and 5th-year students was 59.27%, 56.56%, and 62.7%, respectively. The ANOVA test revealed statistically significant differences, as the p-value of 0.01 was less than 0.05. For the GPA variable, the mean percentage of correct answers for GPA groups <3.5, 3.5 to 3.99, 4 t 4.49, and 4.5 to 5 was 45.24%, 63.99%, 57.55%, and 61.45%, respectively.

**Table 1:** Demographic distribution and analytical results GPA: Grade point average, SD: Standard deviation, CI: Confidence interval

Variables		Number of participants (%)	Mean percentage of correct answers (SD)	95 % CI Of the mean	p-value
Gender	Female	112 (52%)	59.99 (12)	57.74 - 62.24	0.684 (t-test)
						Male	103 (48%)	59.36 (12.3)	56.91 - 61.72
	Academic year	3rd year	74 (34%)	59.27 (12.1)		56.47 - 62.06	0.01 (one-way ANOVA)
4th year		65 (30%)	56.56 (11.1)	53.81 - 59.30			
					5th year	76 (35%)		62.7 (12.5)	59.87 - 65.57
					GPA	4.5 to 5		83 (39%)	61.45 (12.4)	58.74 - 64.15	0.001 (one-way ANOVA)
4 to 4.49		94 (44%)	57.55 (10.6)	55-37 - 59.72							
3.5 to 3.99	32 (15%)	63.99 (13)	59.29 - 68.68								
				<3.5		6 (2%)	45.24 (11.6)	33.09 - 57.37			
				Total		215	59.67 (12.1)	58.04 - 61.30			

Table 2 shows the distribution of answers in each element of the questionnaire.

**Table 2:** Answer distribution among participants

N	Item	True	False	I don’t know
1	Professionalism is exclusive to the medical profession.	6.9	90.3	2.8
2	Professionalism means to be an expert in the profession (being an expert means having knowledge and skills).	36.6	57.9	5.6
3	Professionalism refers to methods of performance in medicine.	43.5	45.4	11.1
4	Professionals do not seek money.	41.7	48.6	9.7
5	Being professional requires academic education and specialized training.	56.9	33.8	9.3
6	Being active in a profession requires a professional organization (any profession requires a professional organization).	56	21.8	22.2
7	Professionals shape the ethical principles of their job themselves.	57.9	25.9	16.2
8	The ethical principles and professional codes are developed by an organization outside their guild and profession.	32.4	34.3	33.3
9	Professionalism is rooted in the theory of deontological ethics.	36.1	9.3	54.6
10	A professional is one who is exclusively involved in one profession full-time.	21.3	66.2	12.5
11	A central element in professional activities is to serve the people and provide them with services.	75.5	9.7	14.8
12	A professional is always accountable for his actions no matter what the circumstances.	69.9	19	11.1
13	Codes of ethics are essential for organizing a profession.	88	3.7	8.3
14	If one fails to adhere to the codes of ethics, one will be reprimanded by the professional guild.	50.5	10.6	38.9
15	A central element in professionalism is the “ethical actor” or “ethical agent.”	63.9	7.4	28.7
16	The doctor-patient relationship is a central element of professionalism	93.3	2.7	4
17	Professionalism focuses on physicians’ personalities.	34.3	47.7	18.1
18	Excellence is one of the most important obligations of a professional (professional excellence is to be involved in continuous education and update one’s knowledge in theory and practice).	77.3	6	16.7
19	Professionalism is rooted in the theory of virtue ethics.	56	10.2	33.8
20	Only doctors are considered professionals.	4.2	93.5	2.3
21	Professionalism is an issue that was only proposed in medicine 20 years ago.	20.8	22.7	56.5

## Discussion

Professionalism is a broad concept that may be affected by many determinants. Some possible determinants are environmental, such as culture, and some are related to personal traits and characteristics, such as gender and age. This study explored several demographic characteristics, such as gender, academic level, and academic performance (GPA). 

The results revealed that males and females had similar scores for knowledge levels, as statistical tests showed no statistically significant differences between males and females. Similar scores between males and females could reflect a collaborative educational environment in the educational institution [12]. A similar study conducted in Iran evaluated the knowledge of professionalism [1] and showed statistically significant differences between males and females regarding knowledge of professionalism. The inconsistent results between the two studies could be due to different educational environments and cultural factors in both countries. However, several studies evaluating other aspects of professionalism rather than knowledge of professionalism [7,12] showed similarities and no difference between males and females. 

Among the participants, 5th-year students achieved the highest mean percentage of correct answers in the test, followed by the 3rd and 4th-year students (62.7%, 59.27%, and 56.56%, respectively). The differences between the academic year groups were statistically significant. The results correlate with the expectation that professional development evolves in parallel with acquiring basic science and clinical knowledge [7]. Third-year students exhibited higher scores than 4th-year students, probably because a curriculum focused on professionalism was implemented throughout the second academic year, which may justify this finding and the recall bias of 4th-year students. A similar study reported that students’ attitudes toward professionalism tended to deteriorate during their years of training [13].

Since this study was conducted to evaluate the knowledge of professionalism, it was crucial to correlate it with academic performance. Therefore, the GPA of the students was obtained and analyzed in the form of class intervals (<3.5, 3.5 to 3.99, 4 to 4.49, and 4.5 to 5). The results showed a statistical difference in the GPA groups, indicating that students with higher GPAs had higher knowledge scores. Except for the class interval of 3.5 to 3.99, where students exhibited the highest knowledge scores for professionalism, this was probably due to non-response bias since the GPA variable was not fixed by a stratified random sample in this study. A similar study conducted to assess the correlation between professional behavior and academic performance showed similar results and demonstrated a positive correlation between professional behavior and academic achievement [14]. 

In comparison with a similar study conducted in Iran at the Shahid Beheshti University of Medical Sciences [1] to evaluate the knowledge of medical professionalism using the same assessment method as this study, the mean percentage of correct answers by males and females was 47.67% and 51.87%, respectively. However, the mean percentage of correct answers among participants in the current study for males and females was 59.36% and 59.99%, respectively. Therefore, the mean percentage of correct answers in the current study was higher than in the other study, possibly due to the presence of teaching courses on professionalism, as the participants in the other study were not taught or assessed about professionalism as part of the medical curricula in their college, unlike the participants in the current study, who have a focused curriculum in college. This supports the evidence that a focused curriculum in professionalism may improve participant knowledge and the overall behavior of the group [11]. A multi-regional study evaluating cultural similarities and differences in medical professionalism showed regional similarities and dissimilarities in understanding professionalism, which may justify the differences between the two studies [15]. 

Limitations 

This study only evaluated the knowledge of medical professionalism among medical students and excluded other crucial aspects, such as attitudes or behaviors. Also, we excluded many other factors due to a lack of feasibility. Despite the validity of the assessment tool, there was no certainty whether this questionnaire reflected the true awareness of professionalism among responders. The study results cannot be generalized across the medical community because the study included only medical students (3rd, 4th, and 5th years) from one college. 

## Conclusions

In conclusion, a highly significant association was found between knowledge of professionalism and both academic level and performance among medical students. This suggests that professional perception evolves along with the learning of basic science and clinical knowledge. Identifying the correlation between knowledge of professionalism and the possible determinants helps develop more effective approaches to promoting this quality in medical education. The study strongly recommends that medical educators worldwide collaborate and share ideas on developing medical professionalism.
